# Clinical and microbiological efficacy of indocyanine green-based antimicrobial photodynamic therapy as an adjunct to non-surgical treatment of periodontitis: a randomized controlled clinical trial

**DOI:** 10.1007/s00784-023-04875-w

**Published:** 2023-01-31

**Authors:** Marco Annunziata, Giovanna Donnarumma, Agostino Guida, Livia Nastri, Gerardo Persico, Alessandra Fusco, Ignacio Sanz-Sánchez, Luigi Guida

**Affiliations:** 1 Multidisciplinary Department of Medical-Surgical and Dental Specialties, University of Campania “Luigi Vanvitelli”, Naples, Italy; 2 Department of Experimental Medicine, University of Campania “Luigi Vanvitelli”, Naples, Italy; 3 U.O.C. Odontostomatologia, A.O.R.N. “A. Cardarelli”, Naples, Italy; 4 Etiology and Therapy of Periodontal and Peri-Implant Diseases (ETEP) Research Group, University Complutense, Madrid, Spain

**Keywords:** Photochemotherapy, Periodontitis, Indocyanine green, Diode laser

## Abstract

**Objectives:**

The aim of the present randomized clinical trial (RCT) with a parallel arm design was to evaluate the clinical and microbiological efficacy of repeated ICG-aPDT as an adjunct to full-mouth subgingival debridement in the treatment of periodontitis.

**Materials and methods:**

Twenty-four periodontitis patients were treated with full-mouth ultrasonic subgingival debridement (FMUD). Initial sites with probing depth (PD) > 4 mm were randomly assigned to receive the test (ICG-aPDT with an 810 nm diode laser) or the control treatment (off-mode aPDT) one and four weeks after FMUD. Clinical parameters were registered after 3 and 6 months. The presence of the main periodontal pathogens in subgingival samples was assessed with real-time PCR.

**Results:**

Both treatment modalities resulted in significant clinical improvements at 3 and 6 months. The only significant differences in favour of the test group were found at 6 months for a higher PD reduction in initial deep pockets (PD ≥ 6 mm) and a higher percentage of closed pockets (PD ≤ 4 mm/no bleeding on probing). Limited microbiological changes were observed in both groups after treatment with no inter-group difference, except for a more significant reduction in *Aggregatibacter actinomycetemcomitans* and *Parvimonas micra* levels in the test group at 3 months.

**Conclusion:**

The combination of repeated ICG-aPDT and FMUD provided no benefits except for selective clinical and microbiological improvements compared to FMUD alone.

**Clinical relevance:**

Based on the obtained results, only limited adjunctive effects could be found for the combined use of ICG-aPDT and FMUD. Further, well-designed RCT with larger sample sizes are required to confirm these findings.

**Trial registration:**

ClinicalTrials.gov NCT04671394.

**Supplementary Information:**

The online version contains supplementary material available at 10.1007/s00784-023-04875-w.

## Introduction

Periodontitis is a chronic multifactorial inflammatory disease initiated by a dysbiotic biofilm and mediated by a dysregulated host response. It is characterized by the progressive destruction of the supporting periodontal tissues [1] and, if untreated, can lead to tooth exfoliation, with possible severe functional and aesthetic impairments [2]. The bacteria most associated with the aetiology of this disease are Gram-negative anaerobic species, some of which can invade the gingival tissues, such as *A. actinomycetemcomitans* and *P. gingivalis*, and are probably associated with the most severe and rapidly progressive forms of periodontitis [3]. In the majority of cases, periodontitis is preventable and treatable according to a pre-established stepwise approach [4]. A central role in periodontal therapy is played by cause-related therapy, which is aimed at controlling (reducing/eliminating) the subgingival biofilm and calculus by manual and/or power-driven subgingival instrumentation [5, 6]. In addition to subgingival instrumentation, adjunctive interventions have been suggested, including the use of physical or chemical agents [7], local or systemic host-modulating agents [8], and antimicrobials [9, 10]. The effectiveness of non-surgical therapy, in fact, can be affected by limited access to certain sites, such as deep pockets, furcation areas, concavities, grooves, or distal sites of molars, which may impair periodontal healing due to the persistence of pathogens and subsequent recolonization [11, 12].

In this context, the use of lasers in combination with photosensitizer solutions, namely, antimicrobial photodynamic therapy (aPDT), has been proposed as an adjunct to conventional periodontal therapy to enhance the antibacterial effect and, thus, to improve clinical results especially in areas with difficult access. The goal of aPDT is to use a photosensitizer solution that once absorbed by bacteria is activated with a light irradiation, thereby producing reactive oxygen species, which injure bacteria without the risk of developing bacterial resistance [13].

Due to the heterogeneity of proposed protocols and the conflicting results on their efficacy for the treatment of periodontitis, the evidence for adjunctive aPDT is as yet insufficient for clinical recommendation [4]. However, a growing interest in such a therapeutic approach exists and novel combinations of lasers and photosensitizers are constantly being investigated.

The combined use of diode laser and indocyanine green (ICG) has been recently proposed for the adjunctive treatment of periodontitis. ICG is approved for use as a fluorescence perfusion dye and as a photosensitizer. In addition to oxidative attack, it has been reported to exert, in response to near-infrared lasers, a photothermal effect that causes cell injury related to the hyperthermic condition and deeper light penetration [14, 15]. In a recent systematic review on the adjunctive use of ICG-aPDT in the non-surgical treatment of periodontitis, significant improvements in clinical outcomes have been reported. Nevertheless, the authors emphasized the need for more high-quality RCTs to draw specific clinical recommendations [16].

Given the relevance of this topic and the existence of limited and heterogeneous data in the literature, the aim of the present randomized controlled trial (RCT) was to evaluate the clinical and microbiological adjunctive effects of repeated applications of ICG-aPDT as an adjunct to FMUD in the treatment of periodontitis.

## Materials and methods

### Study design and general information

The study was designed as a 6-month, single-blinded, parallel group RCT with a 1:1 allocation ratio and was reported according to the CONSORT statement (http://www.consort-statement.org/). It was conducted at the Periodontology and Implantology Unit of the University of Campania “Luigi Vanvitelli” (Naples, Italy), whose Institutional Review Board approved the study protocol (Ref. n. 525/2014). This study was conducted in accordance with the Helsinki Declaration of 1975, as revised in 2013. All voluntary participants were informed of the outline, purpose and duration of the study and signed an informed consent form. This study was registered on clinicaltrials.gov (ID: NCT04671394).

### Sample size calculation

Sample size calculation determined that 10 subjects per treatment group would provide an 80% power to detect a true difference of 1.0 mm between the test and control groups using probing depth (PD) reduction as the primary outcome variable. Assuming that the common standard deviation would be 0.8 mm and compensating for a 20% drop-out during the study period, a sample of 12 subjects per group (24 in total) was determined.

### Patient recruitment

After a screening visit including a full-mouth periodontal and radiographic evaluation, all patients fulfilling the following criteria were asked to participate.

The inclusion criteria were as follows:

• Age between 18 and 80 years

• Systemically healthy

• Diagnosis of chronic periodontitis based on the presence of at least 4 teeth per quadrant with PD>4 mm and radiographic bone loss between 30 and 50% in more than 30% of teeth [17]. Based on the new classification [18], these patients would be diagnosed as generalized stage II–III periodontitis

The exclusion criteria were as follows:

• Systemic diseases requiring antibiotic prophylaxis or other systemic medication that could affect the patient’s clinical response

• Periodontal treatment within the last 12 months or systemic antibiotic intake in the last 3 months

• Pregnant women or those planning to get pregnant in the next 6 months

### Outcome variables

One blinded and calibrated examiner, different from the operator, performed all measurements. This examiner, before beginning the study, carried out a calibration session on five randomly selected patients, resulting in mean differences between repeated measurements of 0.5 mm for PD, with an intra-examiner reproducibility of 98% and 81% for differences ±1 and ±0.5 mm, respectively. The clinical measurements were performed in all teeth at 6 sites per tooth at baseline, 3 and 6 months. The changes in the following outcome variables were calculated:

1. Primary outcome

• PD, defined as the distance between the gingival margin (GM) and the bottom of the periodontal pocket. PD was recorded with a manual probe (UNC-15, Hu-Friedy, Chicago, IL USA) using a light force and measured to the closest millimetre.

2. Secondary outcomes

• Recession (REC), defined as the distance between the GM and the cemento-enamel junction (CEJ) or the margin of the restoration.

• Clinical attachment level (CAL) calculated as the distance between the CEJ and the bottom of the periodontal pocket.

• Bleeding on probing (BOP), defined as the percentage of sites positive to bleeding within 10 s after probing (%).

• Plaque index (PI), defined as the percentage of sites with plaque on the tooth surface (%).

• Patient-reported outcome measures (PRPMs), which include the incidence of adverse effects, and the assessment of intraoperative and postoperative discomfort by means of questionnaires.

### Treatments

Patients were randomly assigned to the test or control group according to a computer-generated list without any restriction. Allocation concealment was assured by opaque, sealed envelopes prepared and sequentially numbered by a person not otherwise involved in the study. Each patient enrolled in the study was instructed to brush and to perform interdental cleaning once daily with interdental brushes. These instructions were reinforced at the re-evaluation visits depending on the plaque scores.

At the first visit (day 0), all patients received a FMUD. The piezo-ceramic ultrasonic device (EMS, Electro Medical Systems, Nyon, Switzerland) with dedicated tips (Piezon A, P, PS, EMS, Nyon, Switzerland) was used under profuse water irrigation with power settings between 50 and 80% for 45–90 min. One week later (day 7), patients were randomly allocated to the test or to the control group, with patients not aware of the assignment group. In the test group, sites with initial PD >4 mm were treated with aPDT using a diode laser device and a photosensitizer solution. In particular, pockets were irrigated by a syringe loaded with an indocyanine green photosensitizer solution (Emundo®, Sweden&Martina, Due Carrare, Padova, Italy) at a concentration of 1 mg/ml. The photosensitizer solution was allowed to stay in the pockets for 2 min, and excess solution was washed out from the pockets before laser irradiation. Afterwards, a 300-μm bulb optical fibre of the 810 nm diode laser unit (Fox ARC, Sweden & Martina, Due Carrare, Italy) set at 300 mw in pulsed mode (100 ms ON/100 ms OFF) was inserted along the pocket and activated for 30 s with continuous vertical movements from the bottom of the pocket to the gingival margin. The patients in the control group were treated by the same operator and received the same treatment, irrigating the pockets with the photosensitizer solution and carrying inside the optical fibre with the laser kept it turned off mode. In both treatment groups, local anaesthesia was only applied when requested by the patient. The same treatment was repeated 3 weeks later (day 28) in both test and control groups. After completing the treatment phase, all teeth were polished supragingivally with a rubber cup and a low abrasive polishing paste (Nupro, Dentsply-Sirona, USA) at each follow-up visit. If during the follow-up period, any patient showed attachment loss of ≥2 mm in ≥4 teeth, or the presence of a periodontal abscess, he or she was excluded from the study and treated again with standard periodontal therapy. Data from these patients were analysed as if they had been dropped for other reasons (intention-to-treat analysis).

### Microbiological analysis

One site per quadrant with the deepest PD and BOP was selected for the microbiological analysis. Subgingival plaque samples were collected at baseline and 3 and 6 months after treatment. After removing the supragingival plaque, the selected sits were isolated from the saliva by cotton rolls and gently dried with air flow [19]. Two sterile paper points (Maillefer, Ballaigues, Switzerland) were consecutively inserted subgingivally and kept in site for 10 s. Afterwards, they were transferred into a sterile vial containing 1.5 ml of reduced transport fluid (RTF) [20] and sent to the laboratory within 2 h.

#### Genomic DNA extraction

The phenol-chlorophorm method was used to extract the bacterial DNA from the samples. Briefly, bacteria were resuspended in Tris-HCl 50 mM pH 8.0, 0.25 M sucrose and 25 mg/ml lysozyme and incubated for 1 h at 37°C. Afterwards, a tungsten carbide bead in sample disruptor (Tissue Lyser, Qiagen, USA) followed addiction of SDS 0.1% was used to lyse the bacteria. The solution was centrifuged, and the aqueous layer was aspirated into a new tube with an equal volume of phenol, which was mixed and centrifuged for 2 min at the highest speed. Again, a new tube was used to transfer the aqueous phase, which was mixed with an equal volume of Tris-saturated phenol/chloroform/isoamyl alcohol (25:24:1). After centrifugating it, a new tube was used to mix the aqueous layer from the previous tube and an equal volume of chloroform, which was centrifugated at the highest speed for 2 min at 4°. Sodium acetate 0.3 M and 2 volumes of ethanol were added to the aqueous phase retrieved from the last centrifugation, which was incubated overnight at −20 °C. One day after, the genomic DNA was precipitated with ethanol 70% and resuspended in water.

#### Real-time PCR

Real-time PCR was used to assess the detection of the following periodontal pathogens within the subgingival samples: *Porphyromonas gingivalis*, *Prevotella intermedia*, *Prevotella nigrescens*, *Campylobacter rectus*, *Aggregatibacter actinomycetemcomitans* and *Parvimonas micra*. The full protocol has been previously published [21]. In summary, the LC Fast Start DNA Master SYBR Green kit was used using 10 ng of DNA in a 20 μL final volume, 3 mM MgCl2 and 0.5 μM sense and antisense primers (supplementary Table s1). The sample was heated to 95° for 15 s (rate of 20 °C/s) after amplification to be able to perform the melting curve analysis. Then, it was cooled to 60 °C for 15 s (rate of 20 °C/s), and finally, it was heated again to 95° (0.1 °C/s). The LightCycler software (Roche Diagnostics) was used to analyse the results. Serial dilutions of cDNA were prepared to calculate the standard curve of each primer pair. All PCR reactions were run three times. Electrophoresis on a 2% agarose gel was performed to verify the specificity of the amplification products, which was visualized with an ethidium bromide staining.

### Statistical analysis

The primary outcome variable was considered to be the change in PD. The percentage of closed pockets (PD < 4 mm and BOP-) was also calculated. PD was stratified in shallow to moderate (initial PD < 6 mm) and deep (initial PD ≥ 6 mm) pockets. Discrete variables (reported as percentages of all sites) were analysed by the Fisher exact test. Continuous variables (reported as means and standard deviations) were compared by the Mann–Whitney *U* test. All comparisons were analysed using two tails and a significance level of 0.05.

## Results

**Fig. 1:**
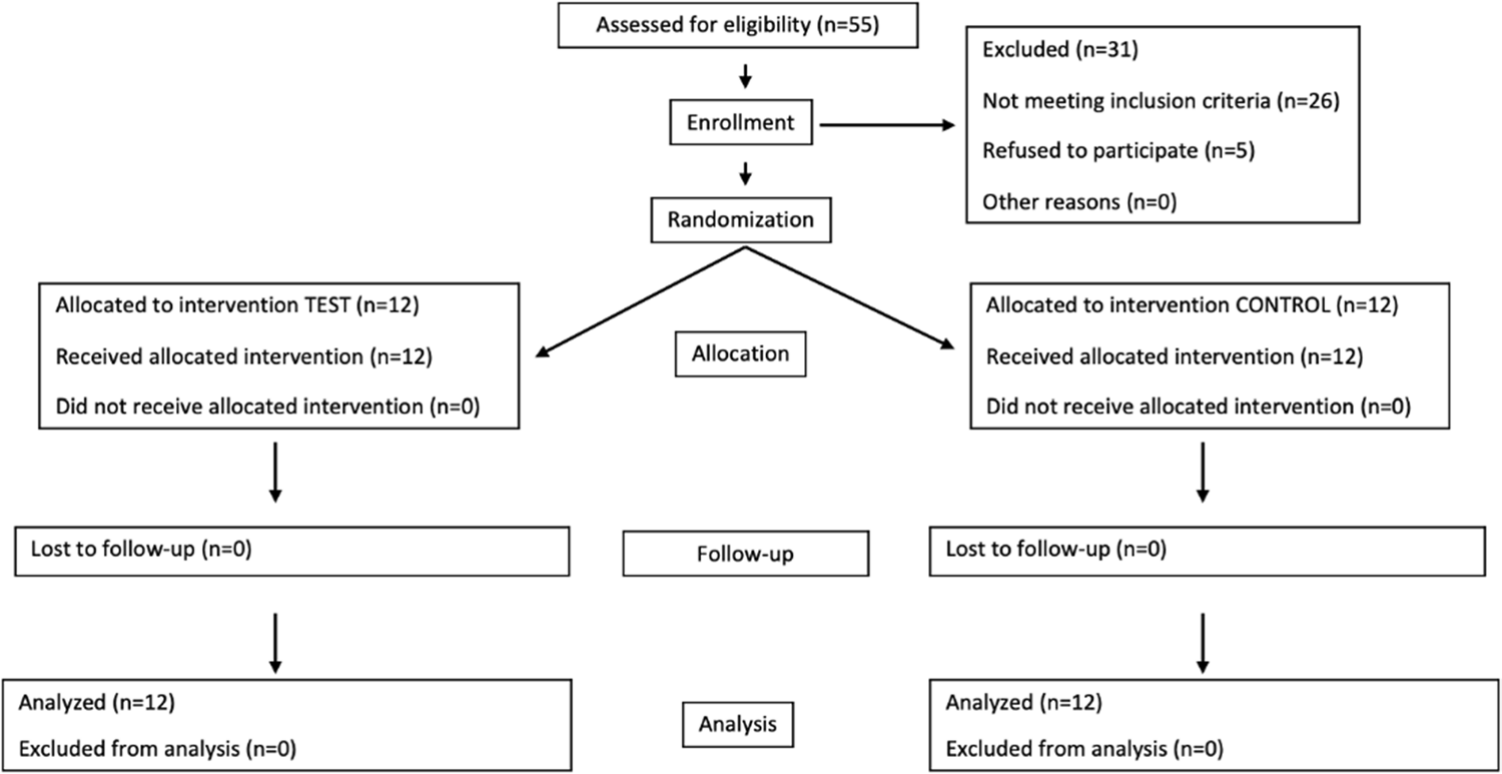
CONSORT flow diagram of the study

Fifty-five patients were consecutively screened, and 24 of them were enrolled, and 12 allocated to the test and 12 to the control group. All patients attended the 3- and 6-month follow-up visits. Figure 1 depicts the CONSORT flow diagram of the study.

The characteristics of the included patients at baseline are listed in Table 1. There were no significant inter-group differences regarding age, gender, proportion of smokers or number of teeth. Taking into consideration the new classification system of periodontal diseases [18], all patients presented stage III, grade B, generalized periodontitis.

**Table 1:** Characteristics of the enrolled patients at baseline

	Test group	Control group	Total
Number of patients	12	12	24
Median age (range)	54 (39, 71)	49 (33, 66)	52 (33, 71)
Gender (male:female)	3:9	6:6	9:15
Smoking habit (non-smokers or former smokers: smokers <10 cig/day: heavy smokers ≥10 cig/day)	7:5:0	6:6:0	13:11:0
Median number of teeth (range)	24 (24, 26)	27 (24, 28)	25 (24, 28)

The baseline mean values for all the clinical outcomes are depicted in Table 2. No significant differences between the groups were observed, neither at baseline nor for the changes at 3 and 6 months for any of the clinical outcomes when taking into consideration all the sites together. A significant PD reduction was found in both groups at 3 and 6 months. Although PD reduction was higher in the test group, no statistically significant differences were observed at any time point. Similarly, CAL gains were registered in both test and control groups at 3 and 6 months, without significant inter-group differences at any time point. REC increased throughout the study period without intra- or inter-group significant differences. The test group experienced an additional increase in recession from 3 to 6 months. BOP and Pl significantly decreased at 3 months in both groups and remained stable at 6 months. No differences were seen between the groups for these outcomes.

**Table 2:** Baseline values and follow-up changes measured at all treated sites expressed as mean values (standard deviation)

	Baseline		3 months		6 months	
	*N*	Mean (SD)	*N*	Mean Δ (SD)	*N*	Mean Δ (SD)
PD (mm)						
Test	12	6.32 (0.66)	12	−1.77 (0.77)*	12	−1.81 (1.02)*
Control	12	6.63 (0.85)	12	−1.54 (1.10)*	12	−1.25 (0.99)*
CAL (mm)						
Test	12	6.77 (0.93)	12	−1.29 (0.89)*	12	−1.06 (1.63)*
Control	12	7.06 (0.89)	12	−1.08 (0.90)*	12	−0.77 (0.81)*
REC (mm)						
Test	12	0.56 (0.59)	12	0.48 (0.54)	12	0.74 (0.90)
Control	12	0.44 (0.56)	12	0.46 (0.59)	12	0.48 (0.63)
BOP (%)						
Test	12	93.75 (11.31)	12	−77.08 (19.82)*	12	−72.92 (22.51)*
Control	12	95.83 (9.73)	12	−68.75 (23.44)*	12	−66.67 (40.36)*
PI (%)						
Test	12	84.72 (13.69)	12	−58.33 (32.57)*	12	−52.08 (27.09)*
Control	12	72.92 (29.11)	12	−43.75 (35.56)*	12	−47.92 (36.70)*

Statistically significant differences evaluated by the Mann–Whitney *U* test. *N*, number of patients; *SD*, standard deviation; mean Δ, mean difference from baseline; *PD*, probing depth; *CAL*, clinical attachment level; *REC*, gingival recession; *BOP*, bleeding on probing; *PI*, plaque index

*intra-group statistically significant difference

PD reduction was significantly greater in deep pockets as compared to shallow-moderate pockets at 3 and 6 months only in the test group (Table 3). Furthermore, deep pockets showed a significantly higher mean PD reduction in the test compared to the control group at 6 months. The percentage of closed pockets (PD ≤ 4 mm and BOP-) was significantly greater in the test group at 6 months when taking into consideration all sites together and the initial deep pockets sub-group (Table 4).

**Table 3:** PD reduction for shallow-moderate (initial PD <6 mm) and deep pockets (initial PD ≥ 6 mm) at 3 and 6 months expressed as mean values (standard deviation)

Mean PD reduction (SD)	3 months		6 months	
	Shallow-moderate pockets	Deep pockets	Shallow-moderate pockets	Deep pockets
Test	−1.11 (0.60)	−1.92 (1.04)*	−1.00 (1.12)	−2.00 (1.32)* ^†^
Control	−1.27 (1.19)	−1.62 (1.85)	−0.73 (0.90)	−1.41 (1.71)

Statistically significant differences evaluated by the Mann–Whitney *U* test. *PD*, probing depth; *SD*, standard deviation; *, statistically significant difference vs. shallow-moderate pockets. †, statistically significant difference vs. control

**Table 4:** Percentage of closed (PD ≤ 4 mm and BOP-) pockets for all, shallow-moderate (initial PD < 6 mm) and deep (initial PD ≥ 6 mm) pockets in the test and control groups at 3 and 6 months

% closed pockets	3 months			6 months		
	All	Shallow-moderate pockets	Deep pockets	All	Shallow-moderate pockets	Deep pockets
Test	40	67	34	45*	44	45*
Control	31	45	27	19	45	16

Statistically significant differences evaluated by the Fisher exact test. *, statistically significant difference vs. control

No adverse events were reported, and no significant differences in terms of intraoperative and postoperative discomfort were observed between the groups.

**Fig. 2:**
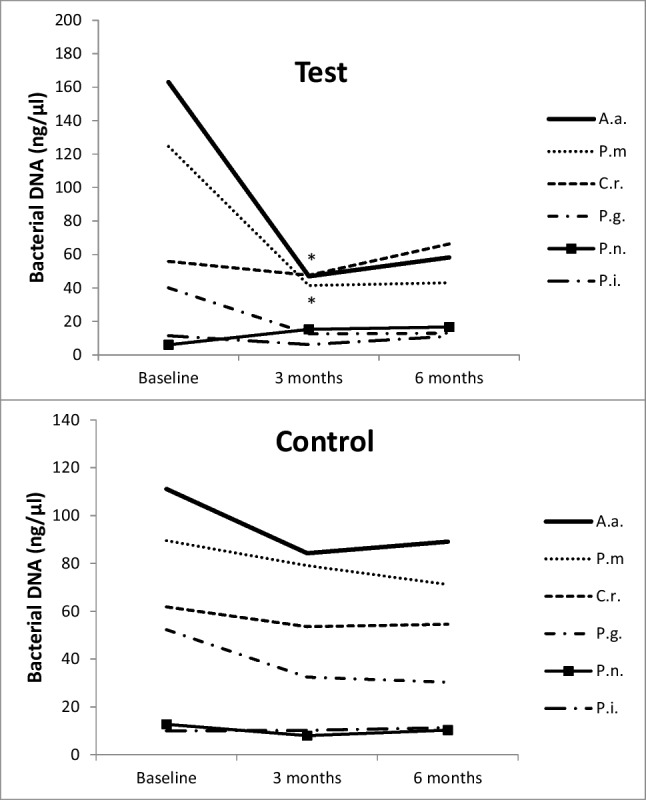
Microbiological results. DNA level of microbial species in test (**a**) and control (**b**) groups at baseline, 3 and 6 months. Statistically significant differences evaluated by the Mann–Whitney *U* test. P.g., *Porphyromonas gingivalis*; *P.m.*, *Parvimonas micra*; C.r., *Campylobacter rectus*; P.i., *Prevotella intermedia*; *P.n.*, *Prevotella nigrescens*; *A.a.*, Aggregatibacter *actinomycetemcomitans.* * = *p* < 0.05

Bacterial species levels showed heterogeneous behaviour from baseline to the follow-up visits (Fig. 2). Some, such as *Prevotella nigrescens* and *Prevotella intermedia*, showed low levels already at baseline, which remained stable at the subsequent time points irrespective of the treatment group. Other species, such as *Porphyromonas gingivalis* (*P.g.*) and *Campylobacter rectus*, showed moderately high concentrations at baseline and were slightly but not significantly reduced after treatment similarly in both groups. On the other hand, *Aggregatibacter actinomycetemcomitans* (*A.a*) and *Parvimonas micra* (*P.m.*) showed high concentrations at baseline in both groups and underwent a significant reduction after treatment at the 3- and 6-month follow-ups. These reductions in *A.a* and *P.m*. were significantly greater in the test group only at 3 months.

## Discussion

The recent EFP S3-level practice clinical guidelines for treatment of stage I–III periodontitis [4] recommend not using an adjunctive aPDT in patients with periodontitis. This recommendation was made on the basis of a systematic review [7] analysing the adjunctive use of a single application of aPDT in the non-surgical treatment of periodontitis, with no significant differences in PD changes at 6 months. It must be emphasized, however, that only five RCTs with a single laser application and a follow-up of ≥6 months were considered, and only two of them (42 patients per group) could be meta-analysed. Furthermore, high variability across the studies was identified in terms of laser type, photosensitizer, wavelength, modality of periodontal treatment, number of treated sites, population and several possible combinations of these parameters. Overall, the available evidence was considered limited and highly heterogeneous, and further, well-designed studies in this area were strongly advocated by the authors.

In line with this recommendation, it was decided to carry out the present RCT. The findings obtained showed only a limited added clinical or microbiological benefit with the adjunctive use of ICG-aPDT, with a significant greater PD reduction at initially deep pockets and a greater increase in closed pockets 6 months after treatment.

In the experimental group, ICG-aPDT was applied only at initial moderate-deep pockets, 1 and 4 weeks after FMUD. This timing was based on an adaptation of the protocol proposed by Wennström et al. [22], in which a second session of full-mouth ultrasonic debridement was carried out 1 week after the first one only at initial deep pockets. This protocol has also been implemented for the adjunctive use of Er-YAG laser in the non-surgical treatment of periodontitis [23, 24]. The rationale for this protocol was to overcome the limitations of ultrasonic instrumentation to completely debride dental biofilm [25] and to reduce inflammation within the pocket to facilitate the action of the laser and the photosensitizer solution. Moreover, this is the first time in which a second round of ICG-aPDT has been applied one month after FMUD with the goal of preventing bacterial recolonization.

Few clinical trials have investigated the adjunctive use of ICG-aPDT to manual and/or ultrasonic debridement in the treatment of periodontitis and with conflicting results. Some investigations reported a significantly higher PD reduction and/or CAL gain in the ICG-aPDT group after 3 or 6 months [26–32], whereas other studies found no significant benefit [33–36]. It must be noted that some methodological differences among these studies exist, such as study population, laser setting regulations, subgingival instrumentation and the laser protocol applied. Therefore, direct comparisons with the present study may be difficult. Two of the above-mentioned studies deserve particular attention [33, 35]. Similar to the present protocol, the authors used FMUD and repeated aPDT applications, with no significant additional benefit of aPDT in terms of relevant mean clinical parameters. However, none of them reported the percentage of closed pockets after treatment. The definition of closed pockets (PD ≤ 4 mm and BOP-) was recently reported also in the EFP S3 level clinical practice guideline for treatment of stage I–III periodontitis [4] where the clinical relevance of this outcome is emphasized, being constantly analysed for the different therapeutic steps together with the mean PD reduction value. Such an outcome, from a clinical perspective, is generally considered more meaningful than PD change mean values [7]. Analysing only the mean PD reduction as a post-treatment outcome, there is the risk of underestimating the real clinical benefit of the therapy and of diverting attention from the real clinical endpoint of the treatment that is pocket closure [4]. In this sense, the present research showed a significantly higher percentage of closed pockets at 6 months in the test group compared to the control group.

Another interesting finding relates to a more relevant effect of aPDT therapy found in initial deep sites, both in terms of mean PD reduction and percentage of closed pockets in the test compared to the control group. In a recent study applying ICG-aPDT [35], the authors highlight how sites with initial deep PD showed higher mean PD reduction compared to all sites together. Furthermore, our finding is in line with other trials [23, 37] in which better clinical results were found applying adjunctive laser therapy in deep periodontal pockets. Such results may sustain the hypothesis of an adjunctive role of aPDT in the non-surgical treatment of sites with difficult access. An increase in PD, in fact, is related to a reduction in the effectiveness of scaling and root planing [11, 12, 25], and aPDT may play a role in potentiating the effects of subgingival instrumentation in deeper sites thanks to the penetration and the activation of the photosensitizer, even in the less accessible areas of periodontal defects. The effects of ICG are thought to be both photochemical and photothermal, enhancing the photothermal effects of high penetration 810 nm diode lasers, thus potentiating their benefits disturbing early bacterial adhesion [38, 39], with a selective effect directly on the dental plaque [40]. Moreover, the near-infrared 810 nm offers additional benefits such as good gingival penetration [41] and mitochondrial activity stimulation [42, 43].

The difficulty in identifying all bacteria belonging to the oral microbiota is mainly represented by the fact that most species are uncultivable, and this makes it necessary to use alternative identification methods. The sequence analysis of 16S ribosomal RNA is currently the most frequently used, thanks to the ubiquitous presence of this polynucleotide in all organisms. Furthermore, through the design of specific primers for PCR-mediated amplification, followed by cloning and Sanger sequencing approach, this method allows describing all the species present in each sample or address specific genera. In the present study, a significant microbiological effect of aPDT was observed for two bacterial species (*A.a.* and *P.m.*) which were significantly reduced in the test group, compared to the control one, after 3 months. However, such an effect was not maintained at the longer follow-up, and no other relevant effects could be observed in terms of bacterial level reduction after both test and control treatments.

There is no consensus on the effect of aPDT on the subgingival microbiota, and the heterogeneity of aPDT and sampling protocols among the studies makes it very difficult to draw definitive conclusions. In the systematic review by Akram et al. [44], 17 RCTs were analysed, concluding that SRP+PDT and SRP presented an equivalent reduction of periodontal pathogens, including *A.a* and *P.g*. Only a few studies specifically addressing ICG-aPDT have investigated the subgingival microbial changes, with conflicting results. Some of them have demonstrated a significantly higher reduction of periodontal pathogens including *A.a* [31] and *P.g.* [28, 31] in patients receiving ICG-aPDT, whereas other studies failed to find significant inter-group differences [29, 33].

Certain limitations of the present study need to be mentioned to enable correct interpretation of the reported findings. Operators, unlike the patients, were aware of the treatment allocation. Nevertheless, in order to reduce possible bias, one calibrated and blinded evaluator, unaware of treatment group, performed all measurements. Moreover, no individual stents were used for periodontal probing, which may have influenced measurement reproducibility. Also only selected sites were treated with the test and control treatments, and a possible cross-influence of other periodontal sites cannot be excluded. Another limitation may be the inclusion of smokers among the patients, due to the noteworthy ability of smoking to affect periodontal treatment outcomes [45, 46], although there was no inter-group difference in the proportion of smokers.

## Conclusions

Taking into consideration the limitations found in the present study, it can be concluded that the adjunctive use of ICG-aPDT to FMUD in initial moderate-deep pockets indicated a limited clinical and microbiological added benefit at 3 and 6 months of follow-up. No significant differences between the groups were observed in the changes of any clinical parameter. However, the test group showed a significantly higher PD reduction in initial deep pockets as well as a significantly higher percentage of closed (particularly deep) pockets at 6 months. Further, RCTs performed on larger patient populations are required to confirm these findings and to investigate the possible factors affecting the clinical efficacy (e.g. number of applications, laser settings) in order to clarify whether there is a rationale for the application of this aPDT protocol in conjunction with FMUD for the treatment of periodontitis.

## Supplementary information

ESM 1 (DOCX 29 kb)

## Data Availability

The data that support the findings of this study are available from the corresponding author upon reasonable request.
