# Supplementary material for: Clinical and microbiological efficacy of indocyanine green-based antimicrobial photodynamic therapy as an adjunct to non-surgical treatment of periodontitis: a randomized controlled clinical trial
Source: Clin Oral Investig. 2023 Jan 31;27(5):2385–94. doi: 10.1007/s00784-023-04875-w (PMC10159973; doi:10.1007/s00784-023-04875-w)
Supplement: Supplementary file 1 — (DOCX 29 kb) [file 784_2023_4875_MOESM1_ESM.docx]

**Table s1.** Sense and antisense primers used for Real-Time PCR.

| **Name of bacteria** | **Primers sequence** | **Amplification programs** | | **Base pairs** |
| --- | --- | --- | --- | --- |
| ***Porphyromonas gingivalis*** | 5’-TGTAGATGACTGATGGTGAAAACC-3’  5’-ACGTCATCCCCACCTTCCTC-3’ | | 10’’at 95°C, 4” at 60°C, 8’’at 72°C for 40 cycles | 197 |
| ***Prevotella intermedia*** | 5’-TTTGTTGGGGAGTAAAGCGGG-3’  5’-TCAACATCTCTGTATCCTGCGT-3’ | | 10’’at 95°C, 11” at 55°C, 23’’at 72°C for 40 cycles | 575 |
| ***Prevotella* *nigrescens*** | 5’-ATGAAACAAAGGTTTTCCGGTAAG-3’  5’-CCCACGTCTCTGTGGGCTGCGA-3’ | | 10’’at 95°C, 16” at 55°C, 32’’at 72°C for 40 cycles | 804 |
| ***Campylobacter* *rectus*** | 5’-TTTCGGAGCGTAAACTCCTTTTC-3’  5’-TTTCTGCAAGCAGACACTCTT-3’ | | 10’’at 95°C, 12” at 60°C, 24’’at 72°C for 40 cycles | 598 |
| ***Aggregatibacter* *actinomycetemcomitans*** | 5’-AGAGTTTGATCCTGGCTCAG-3’  5’-CACTTAAAGGTCCGCCTACGTGCC-3’ | | 10’’at 95°C, 12” at 60°C, 24’’at 72°C for 40 cycles | 593 |
| ***Parvimonas* *micra*** | 5’-AGAGTTTGAATCCTGGCTCAG-3’  5’-ATATCATGCGATTCTGTGGTCTC-3’ | | 10’’at 95°C, 4” at 60°C, 8’’at 72°C for 40 cycles | 207 |
